# Surgical blood loss during holmium laser enucleation of the prostate (HoLEP) is not affected by short-term pretreatment with dutasteride: a double-blind placebo-controlled trial on prostate vascularity

**DOI:** 10.18632/aging.102883

**Published:** 2020-03-11

**Authors:** Gian Maria Busetto, Francesco Del Giudice, Martina Maggi, Gabriele Antonini, Daniele D’Agostino, Daniele Romagnoli, Alessandro Del Rosso, Marco Giampaoli, Paolo Corsi, Katie Palmer, Matteo Ferro, Giuseppe Lucarelli, Daniela Terracciano, Ottavio De Cobelli, Alessandro Sciarra, Ettore De Berardinis, Angelo Porreca

**Affiliations:** 1 Department of Urology, Sapienza Rome University Policlinico Umberto I, Rome, Italy; 2 Department of Urology, Policlinico Abano Terme, Abano Terme (PD), Italy; 3 Department of Internal Medicine and Geriatrics, University Cattolica del Sacro Cuore, Rome, Italy; 4 Department of Urology, Istituto Europeo di Oncologia (IEO), Milan, Italy; 5 Department of Emergency and Organ Transplantation, Urology, Andrology and Kidney Transplantation Unit, University of Bari, Bari, Italy; 6 Department of Translational Medical Sciences, University of Naples Federico II, Naples, Italy

**Keywords:** holmium laser enucleation of the prostate, 5α-reductase inhibitors, vascular endothelial growth factor, microvascular density, dutasteride

## Abstract

Five α-reductase inhibitors (5ARIs) are able to reduce prostate volume and are a useful treatment for reducing perioperative bleeding during prostate surgery. Holmium laser enucleation of the prostate (HoLEP) is an effective surgical technique for the definitive cure of benign prostate enlargement.

We investigated whether pretreatment with dutasteride before HoLEP could reduce intraoperative bleeding. A total of 402 patients were included in this double-blind placebo-controlled trial to receive daily 0.5 mg of dutasteride or placebo over 8 weeks before HoLEP. Vascular endothelial growth factor (VEGF) and microvascular density (MVD) were evaluated. Analysis was also stratified according to prostate volume (<70 mL vs ≥70 mL).

Hemoglobin and hematocrit values before and after surgery were not statistically different between the two groups. MVD and VEGF index in smaller prostates were 23.35±1.96 and 4.06±0.76 in the treatment group and 19.04±0.96 and 2.55±0.55 in placebo (p<0.05); in patients with larger prostates MVD and VEGF were 26.83±2.812 and 8.54±1.18 in the treatment group and 20.76±0.79 and 3.21±0.54 in placebo (p<0.05).

Vascularization of the prostate was affected by 5ARIs therapy. HoLEP is less burdened in perioperative bleeding and for this reason we did not find any difference in hemoglobin/hematocrit values pre- and post- surgery.

## INTRODUCTION

Benign prostate enlargement, with related lower urinary tract symptoms (LUTS), is one of the most common diseases for patients who are referred to a urologist. Benign prostate enlargement strongly impacts quality of life and is accompanied by a substantial economic burden [1–2].

Disease management is initially based on watchful waiting and medical therapy. In particular α-blockers are able to increase International Prostate Symptom Scores (IPSS) by 30-40% and flow Q_max_ by 20-25% inhibiting the effect of released noradrenaline on smooth muscle cells in the prostate, consequently reducing prostate tone and bladder obstructive outlet [1–3]. With a different mechanism of action, 5α-reductase inhibitors (5ARIs) are able to block the conversion of testosterone in its active form, dihydrotestosterone (DHT). There are two different types of 5α-reductase (type 1 and 2); finasteride and dutasteride are two 5ARIs; the first inhibits type 2, while the second inhibits both type 1 and 2. These drugs induce apoptosis of prostate epithelial cells leading to improvement in lower urinary tract symptoms, as well as a 18-28% volume reduction and a 50% decrease in Prostate Specific Antigen (PSA) after a minimum of six months of therapy [4–5]. Compared to finasteride, dutasteride is more effective in reducing DHT: approximately 70% compared with 95% [6]. Although weak evidence suggests a difference in the onset of clinical benefits due to dutasteride versus finasteride, many comparative trials data do not confirm this finding.

Surgery is the most effective treatment for the definitive cure of the disease, particularly in those cases not responding to medical therapy. Trans-urethral resection of the prostate (TURP) is the gold-standard surgical option for lower urinary tract symptoms /benign prostate enlargement but it is burdened by some complications. Intraoperative bleeding and post-surgery anemia with clots retention are common and 2.9% of patients require blood transfusion [7]. Extensive clinical research for a more effective and safer surgical alternative is underway and several minimally invasive techniques have been proposed to overcome common transurethral resection limitations. Minimization of the risk of bleeding and transfusion is always cited as the most important advantage of all new surgical techniques [8, 9].

The holmium:yttrium-aluminium garnet (Ho:YAG) laser is a pulsed system with a wavelength of 2,140 nm obtaining tissue coagulation and necrosis limited to a depth of 3-4 mm while also providing a hemostatic effect [10]. Holmium laser enucleation of the prostate (HoLEP) is an effective alternative treatment to TURP or open surgery. Several meta-analyses report that HoLEP is effective in terms of LUTS relief and improving IPSS score and uroflow parameters at a comparable, or better, level than TURP [11–13]. One of the main advantages of HoLEP is that reduces intraoperative and post-operative bleeding, leading to a lower transfusion rate, shorter hospitalization, and less catheterization [14, 15]. Patients undergoing these procedures are not required to discontinue anticoagulants or antiplatelet drugs [16]. Even if HoLEP is an excellent option for men with very large prostates, it has little or no advantage in smaller prostate glands when compared to other laser therapies [17, 18].

Preoperative therapy with 5ARIs is useful for reducing bleeding during and after TURP; pretreatment with finasteride 2-4 months before surgery can reduce bleeding in large glands due to a vascularization action [19]. A metanalysis reported that blood loss is significantly reduced during and after TURP in patients taking 5ARIs [20]. However, less data is available concerning the effect of pretreatment with finasteride or dutasteride in patients undergoing HoLEP. Initial reports suggest that they are less successful than for traditional transurethral surgery; microvascular density (MVD) decreases significantly but perioperative bleeding does not differ from control groups [21–23].

Considering that dutasteride reduces prostatic vascularization, particularly in patients with larger prostates, and assuming that HoLEP is an effective and safe surgical procedure for enlarged glands, the current study investigated whether 8 weeks of pretreatment with dutasteride before HoLEP could reduce intraoperative bleeding. The specific aims were to 1) analyze blood loss during and after HoLEP surgery in patients undergoing a short pretreatment with dutasteride compared to placebo, 2) assess whether results differed after stratifying for prostate volume, and 3) evaluate differences in vascular endothelial growth factor (VEGF) and cluster of differentiation 34 (CD34) in morcellated prostatic tissue, a typical immunohistochemical marker of MVD.

## RESULTS

A total of 402 patients were enrolled but 22 were excluded for not meeting the inclusion criteria, leaving 380 patients (Placebo-Group n= 190, Treatment-Group n=190). Patient characteristics are listed in Table 1. A statistically significant difference between the two groups was observed for DHT, PSA, and enucleation rate, while total testosterone and prostate volume were equal. All patients underwent HoLEP and the mean operative time was 69 ± 31 minutes in the Placebo-Group and 76 ± 36 in the Treatment-Group (p = 0.29). An enucleation rate of 1.32 ± 0.34 in the Placebo-Group and 1.09 ± 0.31 in the Treatment-Group (p < 0.05) was obtained; morcellation rate were 5.65 ± 1.98 in placebo group and 5.34 ± 1.91 in Treatment-Group (p = 0.44). Unexpectedly our surgeon noticed a major difficulty in enucleating prostates in patients taking dutasteride and the mean operative time confirms this finding. They found that the space between prostate capsule and adenoma was less demarcated and it was more challenging looking at the correct surgical plane. Furthermore, even though it was not a primary endpoint, a total of 28 incidental prostate cancers were reported after histologic evaluation, 15 in the Group A and 13 in the Group B [24, 25]. Looking at early complications, up to 30 days post-surgery, we found 11 (2.7%) post-operative bleeding and all of these patients required hospital re-admission; in 7 (1.7%) cases a blood transfusion was performed. Late complications (more than 30 days after surgery) reported were: bladder neck stricture in 27 (6.7%) cases and urethral stricture in 13 (3.2%). These patients were treated with catheterization and surgical revision.

**Table 1:** Patients characteristics.

	**Group A (placebo)**	**Group B (dutasteride)**	**p value**
**Patients, n**	190	190	
**Age, y**	60 (53-78)	62 (50-80)	0.49
**PSA, (ng/ml) ± SD**	5.18 ± 4.09	2.79 ± 2.22	< .05
**TT, (ng/dl) ± SD**	599 ± 244	546 ± 313	0.09
**DHT, (ng/dl) ± SD**	51 ± 29	6 ± 5	< .05
**Prostate Volume, (ml) ± SD**	71.55 ± 35.86	68.34 ± 43.37	0.34
**Cut volume, (ml) ± SD**	31.58 ± 9.87	32.15 ± 8.36	0.58
**Enucleation rate (g/min)**	1.32 ± 0.34	1.09 ± 0.31	< .05
**Morcellation rate (g/min)**	5.65 ± 1.98	5.34 ± 1.91	0.44
**Operating time, min**	69 ± 31	76 ± 36	0.19
**Incidental prostate cancer**	15	13	

PSA: prostate specific antigen; TT: total testosterone; DHT: dihydrotestosterone; SD: standard deviation.

Postoperative results are reported in Table 2.

**Table 2A:** Preoperative and postoperative patients characteristics (Group A).

	**Preoperative**	**Postoperative**	**p value**
**IPSS score ± SD**	24.4 ± 4.8	10.1 ± 2.9	< .05
**Qmax, (mL/s) ± SD**	10.1 ± 3.1	26.2 ± 6.4	< .05
**PVR, (mL) ± SD**	99 ± 64	28 ± 18	< .05

**Table 2B:** Preoperative and postoperative patients characteristics (Group B).

	**Preoperative**	**Postoperative**	**p value**
**IPSS score ± SD**	26.2 ± 5.1	10.9 ± 3.0	< .05
**Qmax, (mL/s) ± SD**	11.0 ± 3.1	25.5 ± 5.9	< .05
**PVR, (mL) ± SD**	112 ± 70	26 ± 15	< .05

Patients were divided in two subgroups according to prostate volume (smaller < 70 mL and larger ≥ 70 mL). In patients with smaller prostate volume, hemoglobin, before and after the surgery, were 14.00 ± 2.02 - 12.88 ± 1.94 in the Placebo-Group and 14.24 ± 2.15 - 12.99 ± 2.01 in the Treatment-Groups; hematocrit, before and after the surgery, were respectively 43.25 ± 3.90 - 41.98 ± 4.03 in the Placebo-Group and 42.18 ± 4.07 - 41.17 ± 4.17 in the Treatment-Group. In patients with larger volume prostates, hemoglobin, before and after the surgery were respectively 14.14 ± 2.02 - 12.40 ± 1.99 in the Placebo-Group and 14.21 ± 2.31 - 12.52 ± 2.11 in the Treatment-Group; hematocrit, before and after the surgery were respectively 43.02 ± 3.89 - 40.19 ± 4.00 in the Placebo-Group and 42.59 ± 3.43 - 40.25 ± 3.77 in the Treatment-Group. There was no difference in hemoglobin or hematocrit pre- or post-surgery between treatment groups and the results were the same after stratifying for prostate volume (p values always superior to 0.05).

Conversely, results obtained for MVD and VEGF index in prostates < 70 mL, were 23.35 ± 1.96 and 4.06 ± 0.76 in Group A and 19.04 ± 0.96 and 2.55 ± 0.55 in Group B with a statistically significant difference (p < 0.05); in prostates ≥ 70 mL were 26.83 ± 2.812 and 8.54 ± 1.18 in Group A and 20.76 ± 0.79 and 3.21 ± 0.54 in Group B, once again with a statistically significant difference (p < 0.05). All results are listed in Tables 3 and 4.

**Table 3:** Blood parameters and vascularisation; prostate size < 70 mL.

	**Group A < 70 mL (placebo)**	**Group B < 70 mL (dutasteride)**	**p value**
**Hemoglobin before HoLEP, (g/dl) ± SD**	14.00 ± 2.02	14.24 ± 2.15	0.88
**Hemoglobin after HoLEP, (g/dl) ± SD**	12.88 ± 1.94	12.99 ± 2.01	0.76
**ΔHb ±SD**	1.12 ± 0.12	1.15 ± 0.13	
**Hematocrit before HoLEP, % ± SD**	43.25 ± 3.90	42.18 ± 4.07	0.44
**Hematocrit after HoLEP, % ± SD**	41.98 ± 4.03	41.17 ± 4.17	0.21
**ΔHt ±SD**	1.17 ± 0.21	1.33 ± 0.20	
**MVD**	23.35 ± 1.96	19.04 ± 0.96	< .05
**VEGF index**	4.06 ± 0.76	2.55 ± 0.55	< .05

HoLEP: Holmium Laser Enucleation of the Prostate; MVD: microvascular density; VEGF index: vascular endothelial growth factor index; SD: standard deviation.

**Table 4:** Blood parameters and vascularisation; prostate size ≥ 70 mL.

	**Group A ≥ 70 mL (placebo)**	**Group B ≥ 70 mL (dutasteride)**	**p value**
**Hemoglobin before HoLEP, (g/dl) ± SD**	14.14 ± 2.02	14.21 ± 2.31	0.48
**Hemoglobin after HoLEP, (g/dl) ± SD**	12.40 ± 1.99	12.52 ± 2.11	0.49
**ΔHb ±SD**	1.74 ± 0.22	1.69 ± 0.17	
**Hematocrit before HoLEP, % ± SD**	43.02 ± 3.89	42.59 ± 3.43	0.64
**Hematocrit after HoLEP, % ± SD**	40.19 ± 4.00	40.25 ± 3.77	0.77
**ΔHt ±SD**	2.83 ± 0.54	2.34 ± 0.49	
**MVD**	26.83 ± 2.12	20.76 ± 0.79	< .05
**VEGF index**	8.54 ± 1.18	3.21 ± 0.54	< .05

HoLEP: Holmium Laser Enucleation of the Prostate; MVD: microvascular density; VEGF index: vascular endothelial growth factor index; SD: standard deviation.

## DISCUSSION

In the current placebo-controlled trial we found that administering 0.5 mg of dutasteride for eight weeks pre-surgery did not decrease bleeding during or shortly after HoLEP compared to placebo. Specifically, there were no significant differences in hemoglobin or hematocrit level the changes pre- and post-surgery in patients receiving dutasteride versus placebo. However, microvascular density and vascular endothelial growth factor index were lower in patients receiving dutasteride compared to the placebo group (irrespective of prostate volume). This trial was designed because, after interesting results concerning bleeding reduction with 5ARI during TURP and B-TURP, we wanted to see what effect they had on a surgical procedure that is less burdened by intraoperative and postoperative bleeding, such as HoLEP. Results confirmed our hypothesis that there were no changes in bleeding, even in large prostates. However, a statistically significant difference at the level of microvasculature was found, confirming results reported in our previous experience [26, 27].

Previous studies have shown that dutasteride can be an effective medical treatment for reducing bleeding during and shortly after surgery [14, 15, 20]. Furthermore, two previous studies conducted by our group, have reported favorable outcomes with 5ARIs (the first with finasteride and the second with dutasteride) in terms of successful transurethral resection of the prostate bleeding reducers [26, 27].

Inhibitors of 5α-reductase are able to decrease prostatic bleeding because of the activity on androgen-controlled growth factors that are responsible for angiogenesis and vascularization [26]. Many previous studies have described the effect of 5ARIs on prostate surgery bleeding, focusing on different techniques (open vs TURP vs laser) and different 5ARI [8, 19, 26, 28, 29]. Results on bleeding, operative parameters, and vascularization are different and the effect is still not well established. In a recent meta- analysis with 17 RCTs including 1489 patients, the authors concluded that finasteride is effective in reducing bleeding during TURP while results with dutasteride are inconclusive [20]. Data for HoLEP are both limited and conflicting. Bleeding during and after HoLEP surgery was the main parameter evaluated in our trial, but most previous studies included this as a secondary endpoint.

**Figure 1:**
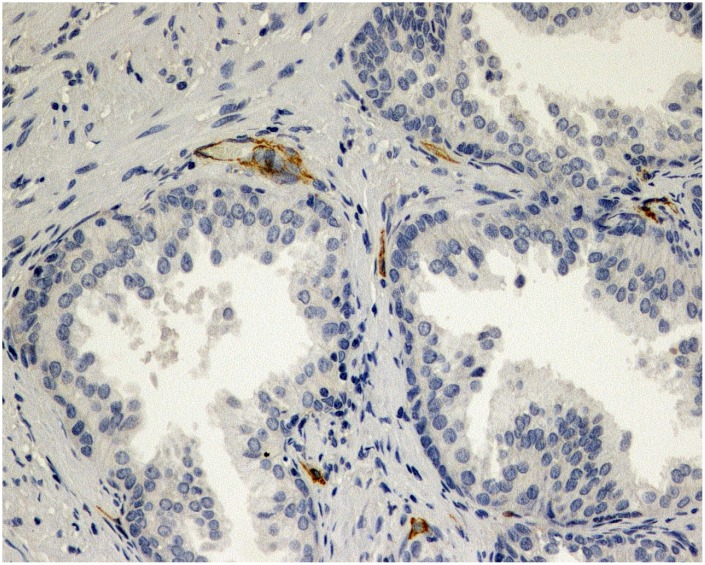
**Immunohistochemical evaluation of prostatic tissue of a patient in placebo group - lower expression of MVD (CD34), brown stained spot coloring vessels (x200).**

**Figure 2:**
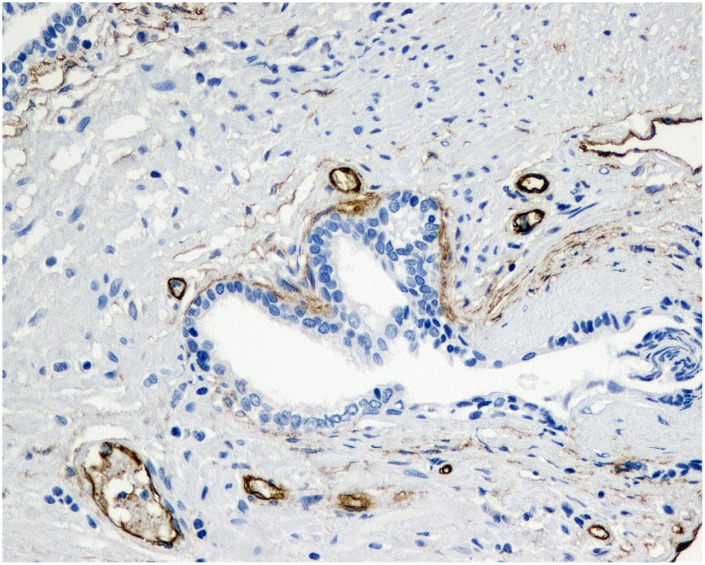
**Immunohistochemical evaluation of prostatic tissue of a patient in 5ARI group - higher expression of MVD (CD34), brown stained spot coloring vessels (x200).**

The present trial also found a difference between the placebo group and patients treated with dutasteride in terms of microvascular density and vascular endothelial growth factor during pathological evaluation following laser prostate enucleation. MVD and VEGF index values were significantly better in those given dutasteride therapy and the pattern was present in patients both with smaller and larger prostate volumes. Another study assessed prostatic tissue of the bladder neck and apex for MVD and reported that pretreatment with dutasteride reduced microvascular density and may lead to decreased risk of perioperative prostatic urethral bleeding [21].

A noteworthy result from our study was that enucleation rates were longer in the patients who took dutasteride. Our experienced surgeon personally reported a major difficulty in enucleating prostates in patients taking this therapy. Another important trial analyzing more parameters has been conducted to evaluate perioperative outcomes of 176 patients after HoLEP. Patients were divided in four groups: no medications, alpha-blocker alone, 5ARI alone, and both 5ARI and alpha-blocker. Enucleation rate was faster in the last two groups, while lower hemoglobin change was reported in favor of the 5ARI group, though this result was not statistically significant. The authors concluded that preoperative 5ARI use does not appear to adversely alter outcomes during HoLEP [23]. The most recent paper is a review on 5ARIs usage before HoLEP that failed to show any particular impact of preoperative 5ARI on surgical efficiency, including bleeding, while surgical difficulty has been reported to be more challenging in the enucleation step for those taking the drug [2].

Trials focused on fibrotic changes of the prostate during 5ARI therapy have confirmed a transforming growth factor-β (TGF-β) concentration increase [30–31]. These fibrotic changes are important because they may cause an increased degree of difficulty in identifying and maintaining the appropriate plane on the surgical capsule of the prostate during transurethral enucleation techniques [22].

The present study had several strengths including the double-blind placebo-controlled design. However, some limitations should be discussed. The patients’ physical features, such as coagulation and body mass index, histological analysis of the resected tissue, surgical reproducibility and quality were not assessed. Furthermore, in our trial tissue fibrotic characteristics were not evaluated. The most important parameter that should have been quantified is blood loss because this could be the best element to exactly measure milliliters of blood lost during the entire procedure. Unfortunately to perform any kind of transurethral surgery (TURP, B-TURP, laser enucleation or laser vaporization) continuous liquid flow is required, which does not allow for a correct measure of blood lost. In addition, there were some differences in baseline characteristics between the placebo and treatment groups.

In recent years, surgical treatment for BPH has been continuously changing and there is an ongoing search for less invasive and more effective approaches. The use of lasers in medicine is well established and is increasingly becoming the gold-standard even for prostate surgery, particularly for big glands [32]. Our trial provides important confirmatory results that that vascularization of the prostate is affected by 5ARIs therapy and MVD together with VEGF index are decreased, confirming this relation. Latest laser surgery techniques are less burdened by perioperative bleeding and we believe that this is the reason that our study found no differences in hemoglobin/hematocrit changes before and after surgery between placebo and dutasteride therapy patients. Finally, a slight difference in surgery difficulty was found after dutasteride therapy.

## CONCLUSIONS

Prostate vascularization is affected by 5ARIs therapy. HoLEP, a surgical technique less burdened by perioperative bleeding, seems to be unaffected by dutasteride preoperative treatment in terms of perioperative and postoperative bleeding.

## MATERIALS AND METHODS

Between January 2016 and December 2018, 402 patients with an average age of 68 (range=50-80) were randomized to receive daily 0.5 mg of dutasteride or placebo 8 weeks before HoLEP. The randomization was: Placebo-Group (Group A) receiving pretreatment with placebo and Treatment-Group (Group B) with dutasteride. Prostate volume was obtained via trans-rectal ultrasound of the prostate the day before surgery and analysis was stratified according to prostate volume (< 70 mL and ≥ 70 mL). Moreover, one hour prior to surgery, a blood withdrawal was carried out to obtain main blood parameters, including hormones. Patients who were taking NSAIDs, anticoagulant, or antiplatelet drugs were asked to discontinue use for a minimum of 7 days prior to surgery (this was done to avoid potential bias, even though this is not mandatory for HoLEP). All surgical procedures were carried out under general anaesthesia by a single experienced surgeon (AP). The surgeon used a 24-French resectoscope (Karl Storz, Tuttlingen, Germany) at low pressure with continuous flow and at the end a 3-way 22FR Dufour catheter (Rüsch Teleflex, Morrisville, NC, USA) was placed. Laser equipment was based on Lumenis Pulse® 100H platform with Lumenis SlimLine™ fibers and morcellator was Lumenis VersaCut™ (Lumenis, Yokneam, Israel). Intravenous hydration was maintained only on the same day as the procedure and maintained constant at 1500 mL (100 mL/hour). Patients were discharged after 36-48h from surgery and at that time another blood test was done.

Inclusion criteria was treatment with α-blockers before HoLEP (discontinued the day before surgery). Exclusion criteria were: patients with renal function impairment (blood creatinine > 145 μmol/l) due to its effect on blood clotting; patients with an alteration of coagulation parameters (blood clotting International Normalised Ratio > 1.3) or any kind of bleeding disorders; patients that, in the past, already underwent any kind of prostate surgery (transurethral or open); patients that already assumed in a different scheme, period, or dosage any kind of 5α-reductase; refusal to sign the informed consent. Taking into account the aforementioned criteria, 22 patients were excluded (see study flow diagram in Supplementary Figure 1).

The aim of the study was to analyze blood loss during and after the surgery, comparing the effect of a short pretreatment with dutasteride or placebo. Changes in vascularization of the prostate were evaluated using vascular endothelial growth factor (VEGF) and microvascular density with the immunohistochemical marker CD34.

The following parameters were registered: hemoglobin and hematocrit before and after the surgery, PSA, total testosterone and DHT. Surgical details were recorded: enucleated prostate volume, enucleation rate per minute, morcellation rate per minute and procedure duration. Morcellated prostate tissue was histologically analyzed and incidental prostatic cancers were recorded. Finally, the material was stained with monoclonal anti-bodies directed against CD34/VEGF, including calculation of CD34 and VEGF index.

Tissue specimens stained with hematoxylin and eosin were fixed in 10% buffered formalin and embedded in paraffin. Sections were cut and slides were dewaxed in xylene and rehydrated through a graded series of ethanols. Heat-induced epitope retrieval was carried out by immersing the slides in citrate buffer (pH 6) and microwaving at 600 W for 20 minutes before rinsing with phosphate-buffered saline (PBS). Endogenous peroxidase activity was quenched by incubating the sections in 1% hydrogen peroxide. Nonspecific binding sites were then blocked by preincubating with 20% normal serum and 1% bovine serum albumin (BSA) in PBS/0.3% Triton X-100 (Union Carbide, Dow Corporation, Midland, MI) for 20 minutes at room temperature. Sections were incubated with polyclonal rabbit anti-VEGF antibody at a concentration of 1:50, as well as monoclonal murine anti-CD34 at a concentration of 1:20. After washing with 0.25% BSA and 0.05% polysorbate 20 in PBS, sections were incubated with biotinylated secondary pan-specific antibody at 1:500 for 1 hour at room temperature. Sections were again washed in PBS with 0.05% polysorbate 20, then incubated with horse radish peroxidase and conjugated streptavidin-biotin complex for 45 minutes. All sections were again washed in PBS with 0.05% polysorbate 20. Immunoreactivity was then visualized by adding hydrogen peroxide as an enzyme substrate, in the presence of 0.05% 3,3′-diaminobenzidine. Nuclei were then lightly counterstained with Harris’s hematoxylin. The area of most intense neovascularization was selected by scanning on low magnification (10-100×), avoiding areas with lymphocytic infiltration or fibrosis. Any brown-staining endothelial cell (CD34-positive) containing a visible nucleus and clearly separate from adjacent microvessels, epithelial cells, and other connective tissue elements, was considered a single, countable microvessel, without requirement for a lumen or the presence of erythrocytes. The microvessels were counted in a 0.74-mm^2^ area. VEGF immunoreactivity was scored for the percentage of stained epithelial glandular and endothelial cells, as 0, 1+, 2+, or 3+, according to staining intensity. A hypertrophic area with any degree of staining was scored as VEGF-positive. Hyperplastic areas that were VEGF-positive and VEGF-negative were assigned the score of the area with strongest staining Figures 1 and 2.

A double-blind placebo-controlled simple randomization was applied. The research staff, surgeon and patients were blind to treatment group. The placebo was administered in the same way as active drug and was made with excipients without the active components.

The statistical analysis was carried out with BioMeDical Package statistical software, version 7 (Statistical Solutions, Saugus, MA) and SPSS (Chicago, IL, version 15.00 for Windows). Statistical significance was achieved if the p-value was < 0.05. All reported p-values are two-sided.

Spontaneously reported adverse events or those noted by the investigator were recorded during the whole study period.

The ethical committee approved the study (registration number 21, obtained on 7^th^ April 2014). All treatments applied were part of routine standard care. The study was conducted in line with European Urology and Good Clinical Practice guidelines, in accordance with ethical principles of the latest version of the Declaration of Helsinki. Every patient signed an informed consent to participate in the study.

## Supplementary Material

Supplementary Figure 1
