# Supplementary figures and images for: Surgical blood loss during holmium laser enucleation of the prostate (HoLEP) is not affected by short-term pretreatment with dutasteride: a double-blind placebo-controlled trial on prostate vascularity
Source: Aging (Albany NY). 2020 Mar 11;12(5):4337–47. doi: 10.18632/aging.102883 (PMC7093193; doi:10.18632/aging.102883)

Flow Diagram

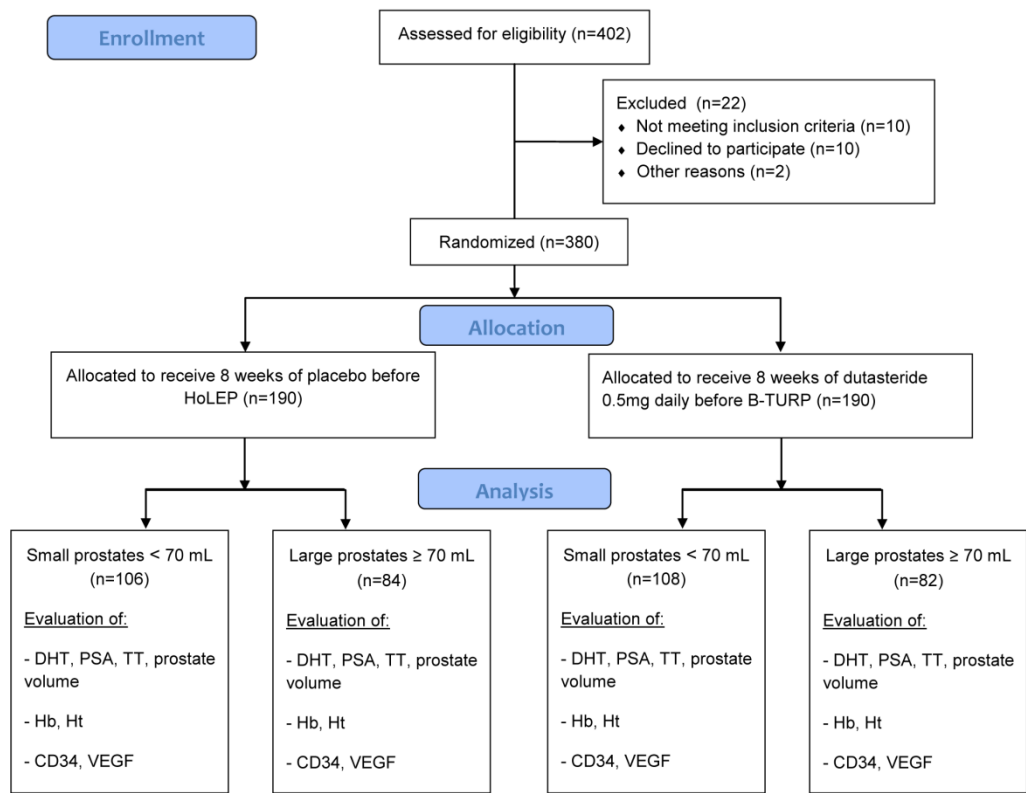

Supplementary Figure 1. Flow diagram of the study.

Supplement: Supplementary Figure 1 [file aging-12-102883-s001..pdf]
